# Intervention Study on the Efficacy and Safety of *Platycodon grandiflorus* Ethanol Extract in Overweight or Moderately Obese Adults: A Single-Center, Randomized, Double-Blind, Placebo-Controlled Trial

**DOI:** 10.3390/nu11102445

**Published:** 2019-10-14

**Authors:** Ye Jin Kim, Eun-Young Kwon, Ji-Won Kim, Youngmi Lee, Ri Ryu, Jongbok Yun, Manheun Kim, Myung-Sook Choi

**Affiliations:** 1 Division of Endocrinology and Metabolism, Department of Internal Medicine, Kyungpook National University, Daegu 41566, Korea; freewilly59@hanmail.net; 2 Center for Food and Nutritional Genomics Research, Kyungpook National University, 1370 San-Kyuk DongPuk-Ku, Daegu 41566, Korea; savage20@naver.com (E.-Y.K.); budy9497@naver.com (J.-W.K.); 6k5rsg@hanmail.net (Y.L.); 3 Department of Food Science and Nutrition, Kyungpook National University, 1370 San-Kyuk Dong Puk-Ku, Daegu 41566, Korea; 4 Research Institute of Applied Animal Science, Institute of Green-Bio Science and Technology, Seoul National University, Pyeongchang 25354, Korea; riryu@anu.ac.kr; 5 Business Deveopment Division, GC WellBeing Corporation, Gyeonggi-do, Seongnam 13595, Korea; jb@greencross.com (J.Y.); caciclup@greencross.com (M.K.)

**Keywords:** *Platycodon grandiflorus* root, BMI, body fat mass, abdominal fat area

## Abstract

*Platycodon grandiflorus* root extract (PGE) has shown various properties, such as anti-hyperlipidemia, anti-diabetic, and anti-obesity, but mostly in animal studies. Therefore, we conducted a preliminary study on the anti-obesity effect of PGE in 108 Korean adults (aged 20–60 years, 30 kg/m^2^ ≥ body mass index ≥ 23 kg/m^2^). The participants were randomly assigned to four groups and were administered the placebo, PGE571 (571 mg as PGE), PGE1142 (1142 mg as PGE), and PGE2855 (2855 mg as PGE), independently, for 12 weeks. Body composition, nutrient intake, computed tomography scan, and plasma adipokines, as well as hepatic/renal function markers, were assessed. The PGE571 group revealed a significant decrease in body fat mass and body fat percentage when compared with the placebo group. Moreover, the total abdominal and subcutaneous fat areas were significantly decreased following PGE (PGE2855 group) supplementation. These results provide useful information on the anti-obesity effect of PGE for overweight and obese adult humans.

## 1. Introduction

Obesity is associated with the morbidity and mortality of diabetes and cardiovascular disease, and it is a major public health problem that is increasing in prevalence worldwide [1]. Obesity increases the risk of metabolic abnormalities, associated insulin resistance, hyperglycemia, type 2 diabetes, and dyslipidemia, which generate high medical costs [2,3]. Obesity leads to the accumulation of body fat mass, as well as the loss of skeletal muscle mass. Recently, a new concept of sarcopenic obesity has emerged, reflecting a combination of sarcopenia and obesity, which describes the process of muscle loss combined with increased body fat as people age—a condition associated with the loss of muscle strength and function, reduced quality of life, and even mortality [4,5].

*Platycodon grandiflorus* (PG) root is widely used in traditional Chinese medicine. It contains many active constituents, such as steroidal saponins, flavonoids, phenolic acids, and sterols, among which, the saponins are regarded as the major active compounds [6]. Numerous studies have proven that the saponins of *P. grandiflorus* exhibit diverse pharmacological activities, such as antioxidant [7], anti-inflammatory [8], and anti-apoptosis effects [9]. These saponins are also believed to have protective effects against some chemically-induced hepatotoxic reactions. Indeed, platycodin D, the major triterpenoid saponin found in PG, was recently suggested for protecting against liver damage caused by ethanol and carbon tetrachloride [10]. In addition, PG is considered to be a legal medicine and dietary supplement; it is also frequently used as an ingredient in health foods and vegetable dishes [11].

Many studies have reported the effects of PG fractions or extracts on lipid metabolism regulation and insulin resistance improvement, but mostly in animal models [11,12,13]. Therefore, we conducted a preliminary study on the anti-obesity effect of PG extract (PGE) in overweight and obese adult humans. This is the first study to investigate the body fat loss effects of PGE in humans. In particular, this preliminary study provides information on the approximate daily dosage of PGE for the upcoming main study, which will be performed using dual-energy X-ray absorptiometry (DEXA) equipment for body fat measurement.

## 2. Materials and Methods

### 2.1. Participants

Volunteers (20–60 years old) were recruited from Daegu and among employees of Kyungpook National University in the Republic of Korea, in March 2017. After an initial screening, 108 participants with a body mass index (BMI) of 23–30 kg/m^2^ were selected. The exclusion criteria were as follows: (1) taking diuretics for hypertension; (2) taking oral hypoglycemic agents or insulin injection; (3) serious cardiac, renal, hepatic, thyroid, or cerebrovascular disease; (4) serious cystic or gastrointestinal disease, gout, or porphyria; (5) psychiatric problems, such as depressive disorder, schizophrenia, alcoholism, and drug intoxication; (6) cancer diagnosis and treatment; (7) asthma or other allergies; (8) a history of surgery within the past 6 months; (9) pregnant or in lactation period. The study was approved by the Kyungpook National University Human Research Committee (KNU 2017-0113). All participants gave their written informed consent for inclusion before they participated in the study.

### 2.2. Sample Size

In order to eliminate the effects of gender differences, the gender of the participants was assigned by replicated randomized complete block design. The sample size was estimated using G* Power 3.1.9.2. Assuming a 95% statistical power, 0.05 significance level, and 0.40 effect size (Cohen’s standard for a large effect), it was estimated that at least 120 participants were required to show a statistically significant difference in biomarkers of body fat among four groups.

### 2.3. Design

This single institution, randomized, double-blinded, and placebo-controlled study was conducted to confirm the effect of PGE supplementation on body fat loss in obese or overweight participants. The random assignment code was generated using the permed-block randomization method with the assistance of SAS Proc Plan (SAS Institute, Cary, NC, USA). All participants were randomly assigned in into four groups in a 1:1:1:1 ratio: placebo, PGE571 (571 mg as PGE), PGE1142 (1142 mg as PGE), and PGE2855 (2855 mg as PGE). The total content of test product for one tablet was 900 mg (including 571 mg of *Platycodon grandifloras* extract); all participants received two pouches per day of the placebo (including 666 mg of crystalline cellulose) and each PGE (PGE571, PGE1142, PGE2855), according to their assigned group, which were consumed from baseline (0 days) to the end of the 12-week experiment, at 30 min after breakfast and dinner. The placebo and PGEs were supplied by GC WellBeing (Seongnam, Korea). All participants were instructed to maintain their routine food intake and physical activity during the study. Moreover, during the study period, we monitored the participants’ compliance with the nutritional intervention and capsule consumption every week by telephone. At the end of the study, the participants were asked to return any pouches not consumed. The doses of PGE were selected by extrapolated calculations on the basis of a previous animal study [14].

### 2.4. Anthropometric and Biochemical Analyses

For anthropometric and physiological measurements at baseline and 4, 8, and 12 weeks post-test material supplementation, the participants visited the Science Research Center Laboratory at Kyungpook National University between 07:00 and 11:00 h after a 12 h overnight fast. The BMI, height, weight, and body composition were measured using an X-Scan Plus II body composition analyzer (Jawon Medical Co., Daejeon, Korea). Abdominal computed tomography (CT; Brivo CT385, GE Healthcare, Chicago, IL, USA) scans that included the lumbar spine were acquired at baseline and during follow-up [2,4]. The CT scans were taken at the Doctors Radiology Clinic, located in Daegu city (Korea). The waist and hip circumferences were measured with an anthropometric tape. The waist circumference was measured as the minimum circumference between the iliac crest and rib cage, and the hip circumference was measured as the maximum width over the greater trochanters. The waist-to-hip ratio was calculated by dividing the waist measurement by the hip measurement. Blood samples were collected in ethylenediaminetetraacetic acid-coated tubes and centrifuged at 1000× *g* for 15 min at 4 °C for plasma assays. To determine the dietary intake, 24 h dietary recalls were administered in face-to-face interviews at the participants’ homes before and during the preliminary trial by dieticians. Three day dietary recalls were performed twice at baseline and during follow-up. We presented the mean of the 3 day dietary intake at each point. During the interview, the participants were asked what kinds of food they ate and drank on their dietary recall sheet. Food replicas were provided to help the participants estimate their dietary intakes and exact portions. Nutritional analysis was performed using CAN-Pro 3.0 software (The Korean Nutrition Society, Seoul, Korea), which provides a comprehensive database for the nutritional content of general foods and specific Korean foods.

### 2.5. Biochemical Analyses

Before and after the test, fasting blood was collected and analyzed by Seegene Co. Ltd. (Daegu, Korea). The levels of plasma adiponectin, leptin, interleukin-6, tumor necrosis factor-alpha, and monocyte chemoattractant protein-1 were determined using a Cobas 8000 analyzer (Roche Diagnostics, Mannheim, Germany). For the safety evaluation of the test material, the liver and renal function markers (albumin, blood urea nitrogen, total bilirubin, aspartate transaminase, alanine transaminase, and alkaline phosphatase (ALP)) were measured using a Hitachi LABOSPECT 008 AS (Hitachi, Tokyo, Japan).

### 2.6. Statistical Analysis

Data were analyzed using SPSS (IBM SPSS, version 21) and expressed as mean ± standard deviation. Statistical analysis was performed using the analysis of covariance (ANCOVA) test for comparison between the placebo control group (placebo) and the test groups. In the questionnaire, it was confirmed that there was a large difference in physical activity and drinking among the participants. Given that there is a high correlation between alcohol consumption and obesity rate [15], participants who frequently consumed alcohol (more than two bottles per week) and reported a very low physical activity (sedentary behavior) were excluded (Table 1). Paired *t*-test was used to verify the difference before and after ingestion at *p* < 0.05. Statistical analysis was also performed on all participants (23 kg/m^2^ ≤ BMI ≤ 30 kg/m^2^) without any discrimination of physical activity and alcohol consumption for safety assessment.

## 3. Results

### 3.1. Study Flow

This study had an initial 130 candidates, and had 108 eligible individuals who were enrolled in this study through the eligibility tests. After 12 weeks, eight people dropped out for personal reasons. Participants with factors affecting obesity, very low physical activity, and frequent alcohol consumption were excluded. Physical activity and alcohol consumption were assessed through the questionnaire (Table 1). Thus, data from 72 participants were analyzed for evaluating the efficacy of PGE supplementation. Serious adverse effects were not reported by the participants consuming the PGEs or placebo supplements.

### 3.2. Baseline Clinical Characteristics and Nutrient Intake

The baseline characteristics of volunteers who completed the randomized controlled trial are shown in Table 2. There were no significant differences among all groups in terms of age, height, systolic blood pressure, diastolic blood pressure, and fasting blood glucose. Nutritional intake of the participants before and after the test food intake was measured six times (three times before the test, three times after the test) using the 24 h recall method. The energy and carbohydrate intake of the PGE1142 and PGE2855 groups were significantly higher than that of the placebo group. In other nutrients, no significant difference was found among all groups (Table 3).

### 3.3. Body Composition

Body composition, such as body weight, BMI, body fat mass, body fat percentage (BFP), and muscle weight, were analyzed by using data that considered drinking and exercise activities (*n* = 72) (Table 4). Body fat (ANCOVA, *p* < 0.028) and BFP (ANCOVA, *p* < 0.001) were significantly decreased in the PGE571 group compared with the placebo. Also, supplementation of PGE2855 led to a decrease in the body fat mass (ANCOVA, *p* < 0.036) and BPF (ANCOVA, *p* < 0.035). In contrast, muscle mass was significantly increased in the PGE571 (ANCOVA, *p* < 0.002)-supplemented group compared with the control (placebo) group (Table 4). 

Moreover, we performed CT scans at baseline and after (follow-up) to observe the effects of PGE supplementation on the participants’ abdominal fat area. The high-dose PGE group (PGE2855) was observed to have a significant effect when compared with the placebo group. In particular, the PGE2855 led to a significant decrease in the L4 total abdominal fat area (ANCOVA, *p* = 0.029) and subcutaneous fat area (ANCOVA, *p* = 0.035) (Table 5).

### 3.4. Plasma Adipokines

Table 6 shows the levels of plasma adipokines. When ANCOVA was used, there were no significant differences in adipokine levels among the four groups. However, PGE571 supplementation significantly reduced leptin levels after 12 weeks from baseline. Also, the leptin:adiponectin (L:A) ratio was significantly lower after supplementation with PGE2855 compared with the baseline (before supplementation) measurement. In comparison to the baseline, PGE supplementation led to a significant decrease in leptin levels in the PGE571 (paired *t*-test, *p* < 0.01) and the L:A ratio in the PGE2855 (paired *t*-test, *p* < 0.05) after the 12 week experiment. In addition, when all participants were analyzed (*n* = 100), adiponectin levels (ANCOVA, *p* < 0.059) tended to decrease in the PGE571 group compared with the placebo (data not shown).

### 3.5. Lipids Metabolism

Table 7 shows the changes in the indirect markers of lipids metabolism. There were no significant changes in lipids metabolism. When all participants were analyzed, there were no significant changes among the four groups in terms of cholesterol, triglyceride, high density lipoprotein cholesterol (HDL-C), low density lipoprotein cholesterol (LDL-C), and phospholipid. The changes were within the normal range and were considered to have no clinical implications in terms of the safety assessment. 

### 3.6. Safety Assessment and Indirect Markers of Hepatic and Renal Function Test

Hepatic and renal function markers in plasma were analyzed for safety assessment. When all participants were analyzed (*n* = 100), there were no significant changes in the safety-related markers of liver or renal function among the four groups. Table 8 shows the changes in the indirect markers of liver and renal function due to PGE supplementation. PGE1142 supplementation was observed to lower the glutamic-pyruvic transaminase (GPT) (ANCOVA, *p* < 0.006) levels more than the placebo. The changes were within the normal range and were considered to have no clinical implications.

## 4. Discussion

Many studies have reported on the health benefits of the extracts and saponin fractions of *P. grandiflorus*, such as anti-obesity and anti-lipid metabolism, but most of them have demonstrated efficacy in animal models. Our preliminary study also confirmed the weight loss and body fat loss efficacy of PGE in animal models of obesity. Indeed, PG extract increased thermogenic gene expression (such as SIRT1, PPARα, UCP1, PGC1α), thereby inducing the browning of white adipose tissue, resulting in an anti-obesity effect. Thus, this study was conducted to verify the effect of PGE in humans. Through the questionnaire, we found that there was a significant difference in the level of alcohol drinking and physical activity among the participants. Accordingly, the following statistics were used in this study: an analysis, excluding dropouts, but including the participants with very low physical activity and frequent drinking activities (more than two bottles per week). 

This study was a small-scale experiment to set the effective dose of PGE in humans by confirming the anti-obesity effect. We conducted bioelectrical impedance analysis (BIA), which is generally highly correlated with body weight and body fat mass, although we did not measure body fat mass by DEXA. 

In particular, we screened participants who frequently consumed alcohol and had a very low physical activity, two factors closely associated with obesity, to confirm the anti-obesity effect of PGE. Energy and carbohydrates were significantly higher in the PGE1142 and PGE2855 supplement groups compared with the placebo. Body composition changes induced by PGE revealed that supplementation of PGE571 and PGE2855 significantly reduced body fat mass and BFP, but also led to an increase in muscle mass in the PGE571 group. In our previous study [14], we found increased muscle mass associated with increased energy expenditure following supplementation of PGE in animal models of obesity. Similarly, in this study, PGE supplementation significantly increased the muscle mass of the participants. Therefore, this increase is considered to be an important candidate in obesity treatment or prevention. We performed abdominal CT, as well as BIA, to confirm the effect of PGE on body fat loss. Consistent with the BIA results, PGE2855 supplementation significantly lowered the subcutaneous fat and abdominal fat area of L4 when compared with the placebo, suggesting the association of BFP and fat mass reduction by PGE2855 supplementation. Although there was no change in the abdominal fat area in the PGE571 group, this may have been due to the relatively high baseline abdominal fat area, as the PGE571 group showed a high level of leptin in the blood, which is proportional to the amount of fat [16]. PGE571 not only significantly decreased body fat and BFP, but also significantly increased muscle mass when BIA was performed, regardless of the statistical method. This was similar to our previous studies that showed increased muscle mass by PGE, which is closely associated with increased energy expenditure [17]. When all participants were analyzed in terms of lipids metabolism and markers of hepatic and renal function test, there were no significant changes among the four groups in terms of cholesterol, triglyceride, HDL-C, LDL-C, phospholipid, and markers of liver and renal function. The changes were within the normal range and were considered to have no clinical implications for the safety assessment. In this context, it is considered that the increase of muscle mass can act as an important marker in obesity (or sarcopenic obesity) research. In this study, DEXA, which is highly correlated with BIA [18] and provides more accurate levels of body fat mass, was not measured. Therefore, it is presumed that DEXA measurement may be able to confirm the change of abdominal fat area due to PGE571 supplementation in future studies. 

The limitations of this trial should be emphasized. Given that the participants of this study were restricted to volunteers, it was difficult to represent the general population with this small sample size. As the study was conducted on overweight or moderately obese adults who had BMI measurements ranging between 23 and 30, part of the results that we obtained via clinical trial were difficult to identify and generate statistically meaningful data. Hence, to have more statistical meaningful data, it would be prudent to conduct the trial in obese adults. This study was a clinical trial to determine whether PGE could improve obesity and estimate the appropriate daily dose of PGE for overweight or obese adult humans. In addition, we used CT scans to improve the accuracy of the body composition measurements, but this technique only measured the abdominal fat area. Therefore, a further experiment is underway to confirm the body fat reduction function of PGE571 using DEXA.

## 5. Conclusions

In conclusion, despite some limitations, we demonstrated that PGE is able to reduce body fat mass and body fat percentage, which is an anti-obesity marker, in overweight or obese adult humans. PGE supplementation significantly increased the muscle mass when compared with the placebo, indicating an excellent anti-obesity effect.

## Figures and Tables

**Table 1:** Survey of physical activity and alcohol drinking status of subjects.

Score	Physical Activity	Drinking Behavior
1	Sedentary behavior	1–2 glasses/week
2	Light activity	Less than 1 bottle/week
3	Moderate activity	1–2 bottles/week
4	Vigorous activity	More than 2 bottles/week

**Table 2:** Baseline characteristics of 4 groups with overweight or obesity subjects who participated in efficacy test of *Platycodon grandiflorus* root extract (PGE).

	Placebo (M: 9, F: 7)	PGE571 (M: 6, F: 13)	PGE1142 (M: 5, F: 14)	PGE2855 (M: 6, F: 12)
Age (years)	40.89 ± 3.44	42.63 ± 2.52	44.95 ± 2.56	48.00 ± 2.03
Height (cm)	168.58 ± 2.79	163.59 ± 2.37	165.26 ± 1.73	162.49 ± 1.82
Body weight (kg)	74.07 ± 2.64	72.48 ± 2.40	70.14 ± 1.93	69.32 ± 1.75
BMI (kg/m^2^)	25.98 ± 0.44	26.99 ± 0.44	25.62 ± 0.41	26.24 ± 0.48
Waist (cm)	93.34 ± 1.21	92.26 ± 1.12	91.76 ± 1.41	90.67 ± 0.84
Hip (cm)	103.00 ± 1.10	101.84 ± 1.00	101.16 ± 0.91	100.64 ± 0.97
WHR	0.91 ± 0.01	0.91 ± 0.01	0.91 ± 0.01	0.90 ± 0.01
Systolic BP (mmHg)	124.69 ± 3.68	133.95 ± 3.95	130.42 ± 3.50	139.11 ± 4.24
Diastolic BP (mmHg)	82.44 ± 2.97	90.21 ± 2.75	85.74 ± 2.27	87.83 ± 2.90

Values are the mean ± SD; PGE571: 571 mg administered as PGE, PGE1142: 1142 mg administered as PGE, PGE2855: 2855 mg administered as PGE, BMI: body mass index, WHR: waist hip ratio, BP: blood pressure, M: male, F: female.

**Table 3:** Comparison of physical activity and nutrients intake in four groups with overweight or obesity by 24 h dietary recall performed before and in a follow-up of the trial.

	Placebo (M: 9, F: 7)	PGE571 (M: 6, F: 13)	PGE1142 (M: 5, F: 14)	PGE2855 (M: 6, F: 12)
	Mean ± SD	Mean ± SD	Mean ± SD	Mean ± SD
Energy (kcal/day)				
Baseline	1571.86 ± 8.31	1680.07 ± 16.89	1520.09 ± 13.74	1747.36 ± 14.15
Follow-up	1384.56 ± 9.83 *	1545.68 ± 14.12	1706.26 ± 15.32	1757.60 ± 10.76
CFB	−187.30 ± 9.45	−134.4 ± 13.21	186.18 ± 12.04	10.24 ± 16.00
*p*-Value		0.437	0.013	0.045
Carbohydrate (d/day)				
Baseline	226.34 ± 1.32	222.70 ± 1.81	221.81 ± 1.85	244.70 ± 1.99
Follow-up	186.23 ± 1.82 **	211.71 ± 2.23	241.07 ± 1.71	245.17 ± 1.80
CFB	−40.12 ± 1.56	−10.99 ± 1.60	19.26 ± 1.89	0.46 ± 2.23
*p*-Value		0.185	0.007	0.021
Fat (g/day)				
Baseline	43.80 ± 0.46	53.95 ± 1.11	43.55 ± 0.68	51.61 ± 0.68
Follow-up	45.17 ± 0.53	48.84 ± 0.57	46.85 ± 0.64	52.97 ± 0.42
CFB	1.37 ± 0.58	−5.11 ± 1.13	3.30 ± 0.52	1.36 ± 0.80
*p*-Value		0.823	0.790	0.358
Protein (g/day)				
Baseline	64.13 ± 0.46	65.61 ± 0.81	57.33 ± 0.55	68.57 ± 0.63
Follow-up	58.72 ± 0.48	57.80 ± 0.55	65.03 ± 0.71	67.96 ± 0.43
CFB	−5.41 ± 0.63	−7.81 ± 0.61	7.70 ± 0.54	−0.61 ± 0.72
*p*-Value		0.804	0.145	0.242
Cholesterol (mg/day)				
Baseline	239.95 ± 3.79	213.90 ± 3.41	203.86 ± 3.40	274.73 ± 3.94
Follow-up	218.46 ± 3.32	234.29 ± 3.71	246.74 ± 3.44	244.84 ± 3.06
CFB	−21.48 ± 4.31	20.39 ± 3.10	42.87 ± 4.83	−29.89 ± 4.20
*p*-Value		0.564	0.341	0.692

*p*-Value: Analysis of covariance (ANCOVA) model with independent variable as baseline and treatment; * *p*  <  0.05, ** *p*  <  0.01 derived from paired *t*-tests performed for values obtained before and after the trial. CFB: changes from baseline.

**Table 4:** Effect of PGE supplementation for 12 weeks on change of body composition measured by bioelectrical impedance analysis (BIA), WHR-related body measurements, and blood pressure in groups with overweight or obesity.

	Placebo (M: 9, F: 7)	PGE571 (M: 6, F: 13)	PGE1142 (M: 5, F: 14)	PGE2855 (M: 6, F: 12)
	Mean ± SD	Mean ± SD	Mean ± SD	Mean ± SD
Body weight (kg)				
Baseline	74.07 ± 2.64	72.48 ± 2.40	70.14 ± 1.93	69.32 ± 1.75
Follow-up	73.61 ± 2.70	72.12 ± 2.42	69.73 ± 1.95	68.03 ± 1.60 **
CFB	−0.46 ± 0.40	−0.36 ± 0.39	−0.42 ± 0.42	−1.28 ± 0.36
*p*-Value		0.901	0.969	0.128
BMI (kg/m^2^)				
Baseline	25.98 ± 0.44	26.99 ± 0.44	25.62 ± 0.41	26.24 ± 0.48
Follow-up	25.71 ± 0.46 *	26.69 ± 0.42	25.39 ± 0.44	25.69 ± 0.46 ***
CFB	−0.27 ± 0.13	−0.30 ± 0.16	−0.23 ± 0.16	−0.54 ± 0.11
*p*-Value		0.933	0.924	0.213
Body fat mass (kg)				
Baseline	21.02 ± 0.95	22.76 ± 0.75	21.91 ± 0.85	22.11 ± 0.98
Follow-up	21.46 ± 0.93	22.12 ± 0.77	21.96 ± 0.91	21.54 ± 0.99 **
CFB	0.44 ± 0.30	−0.64 ± 0.38	0.05 ± 0.30	−0.57 ± 0.20
*p*-Value		0.028	0.432	0.036
BFP (%)				
Baseline	28.73 ± 1.45	31.95 ± 1.18	31.41 ± 1.12	31.97 ± 1.27
Follow-up	29.52 ± 1.42 *	31.11 ± 1.25 *	31.67 ± 1.17	31.73 ± 1.30
CFB	0.79 ± 0.30	−0.85 ± 0.41	0.26 ± 0.30	−0.24 ± 0.17
*p*-Value		0.001	0.273	0.035
Muscle weight (kg)				
Baseline	48.79 ± 2.41	45.61 ± 2.12	44.25 ± 1.68	43.24 ± 1.53
Follow-up	47.94 ± 2.42 **	45.91 ± 2.21	43.82 ± 1.67	42.62 ± 1.43 **
CFB	−0.85 ± 0.26	0.29 ± 0.25	−0.43 ± 0.26	−0.62 ± 0.19
*p*-Value		0.002	0.251	0.551

*p*-Value: ANCOVA model with independent variable as baseline and treatment; * *p*  <  0.05, ** *p*  <  0.01, *** *p*  <  0.001 derived from paired *t*-tests performed for values obtained before and after the trial; CFB: changes from baseline; BFP: body fat percentage.

**Table 5:** Effect of PGE supplementation for 12 weeks on change of abdominal fat area assessed by computed tomography (CT) in subjects with overweight or obesity.

	Placebo (M: 9, F: 7)	PGE571 (M: 6, F: 13)	PGE1142 (M: 5, F: 14)	PGE2855 (M: 6, F: 12)
	Mean ± SD	Mean ± SD	Mean ± SD	Mean ± SD
L4 Total abdominal fat (cm^2^)				
Baseline	305.72 ± 21.54	363.49 ± 15.51	331.80 ± 18.78	318.07 ± 14.08
Follow-up	316.84 ± 19.81	362.35 ± 15.12	331.36 ± 17.47	299.11 ± 11.53 *
CFB	11.12 ± 12.81	−1.13 ± 8.54	−0.44 ± 8.50	−18.95 ± 7.91
*p*-Value		0.959	0.651	0.029
L4 Subcutaneous fat (cm^2^)				
Baseline	184.00 ± 20.17	215.13 ± 14.95	195.32 ± 13.65	179.18 ± 11.76
Follow-up	197.10 ± 18.16	222.43 ± 13.87	196.80 ± 12.28	171.04 ± 10.41
CFB	13.10 ± 11.17	7.30 ± 6.19	1.48 ± 7.15	−8.14 ± 6.73
*p*-Value		0.911	0.382	0.035
L4 Visceral fat (cm^2^)				
Baseline	121.71 ± 7.32	148.35 ± 9.89	136.48 ± 10.37	138.88 ± 11.16
Follow-up	119.74 ± 8.96	139.92 ± 10.52	134.56 ± 10.88	128.07 ± 9.56 **
CFB	−1.97 ± 4.62	−8.43 ± 6.03	−1.92 ± 3.62	−10.81 ± 3.60
*p*-Value		0.561	0.828	0.245

*p*-Value: ANCOVA model with independent variable as baseline and treatment; * *p*  <  0.05, ** *p*  <  0.01 derived from paired *t*-tests performed for values obtained before and after the trial; CFB: changes from baseline.

**Table 6:** Effect of supplementation of PGE for 12 weeks on changes of serum adipokine levels in subjects with overweight or obesity.

	Placebo (M: 9, F: 7)	PGE571 (M: 6, F: 13)	PGE1142 (M: 5, F: 14)	PGE2855 (M: 6, F: 12)
	Mean ± SD	Mean ± SD	Mean ± SD	Mean ± SD
Leptin (ng/mL)				
Baseline	3.58 ± 0.61	6.22 ± 0.96	4.15 ± 0.66	5.47 ± 0.84
Follow-up	3.11 ± 0.46	5.00 ± 0.78 **	4.24 ± 0.62	4.19 ± 0.67
CFB	−0.48 ± 0.42	−1.22 ± 0.35	0.09 ± 0.46	−1.16 ± 0.58
*p*-Value		0.776	0.133	0.888
Resistin (ng/mL)				
Baseline	31.66 ± 8.05	21.83 ± 1.84	19.16 ± 2.57	26.69 ± 4.51
Follow-up	23.65 ± 3.03	21.03 ± 2.79	17.74 ± 2.38	23.17 ± 2.80
CFB	−8.01 ± 6.22	−0.80 ± 2.07	−1.42 ± 2.39	−4.37 ± 2.91
*p*-Value		0.724	0.968	0.726
Adiponectin (ug/mL)				
Baseline	9.74 ± 1.28	8.44 ± 1.61	8.27 ± 1.28	13.53 ± 2.97
Follow-up	9.00 ± 1.37	13.38 ± 4.51	9.84 ± 1.64	11.95 ± 2.16
CFB	−0.74 ± 0.43	4.94 ± 4.60	1.58 ± 1.39	−0.70 ± 1.18
*p*-Value		0.174	0.633	0.792
L:A ratio				
Baseline	0.42 ± 0.07	1.14 ± 0.23	0.84 ± 0.22	0.75 ± 0.22
Follow-up	0.39 ± 0.05	0.74 ± 0.16	0.74 ± 0.20	0.53 ± 0.16 *
CFB	−0.02 ± 0.05	−0.40 ± 0.22	−0.10 ± 0.13	−0.24 ± 0.10
*p*-Value		0.789	0.466	0.758

*p*-Value: ANCOVA model with independent variable as baseline and treatment; * *p*  <  0.05, ** *p*  <  0.01 derived from paired *t*-tests performed for values obtained before and after the trial; CFB: changes from baseline, L:A: leptin:adiponectin.

**Table 7:** Effect of supplementation of PGE for 12 weeks on changes of lipids metabolism in subjects with overweight or obesity.

	Placebo (M: 9, F: 7)	PGE571 (M: 6, F: 13)	PGE1142 (M: 5, F: 14)	PGE2855 (M: 6, F: 12)
	Mean ± SD	Mean ± SD	Mean ± SD	Mean ± SD
Cholesterol (mg/dL): normal range 0~240 mg/dL				
Baseline	201.38 ± 10.42	210.25 ± 9.18	204.43 ± 7.70	198.84 ± 7.30
Follow-up	199.75 ± 10.56	204.55 ± 7.22	208.05 ± 9.69	195.72 ± 8.06
CFB	−1.63 ± 4.34	−5.70 ± 7.40	3.62 ± 5.46	−1.89 ± 5.65
*p*-Value		0.837	0.517	0.904
TG (mg/dL): normal range 36~150 mg/dL				
Baseline	96.00 ± 8.34	119.40 ± 10.81	116.38 ± 9.12	125.79 ± 25.42
Follow-up	119.38 ± 16.70	137.75 ± 15.98	112.00 ± 10.85	116.22 ± 17.78
CFB	23.38 ± 13.33	18.35 ± 11.36	−4.38 ± 8.13	−7.61 ± 21.67
*p*-Value		0.733	0.279	0.324
HDL-C (mg/dL): normal range 40~60 mg/dL				
Baseline	49.88 ± 2.50	53.80 ± 3.06	57.86 ± 2.95	50.11 ± 2.52
Follow-up	46.38 ± 2.49 *	50.95 ± 2.74	56.24 ± 2.23	45.56 ± 2.25 *
CFB	−3.5 ± 1.63	−2.85 ± 2.07	−1.62 ± 1.66	−4.39 ± 1.61
*p*-Value		0.299	0.088	0.704
LDL-C (mg/dL): normal range 0~130 mg/dL				
Baseline	131.69 ± 8.52	134.50 ± 8.47	123.38 ± 6.40	125.68 ± 7.12
Follow-up	130.25 ± 8.01	125.65 ± 6.42	128.38 ± 8.73	124.11 ± 6.50
CFB	−1.44 ± 3.77	−8.85 ± 7.87	5.00 ± 5.02	−0.39 ± 5.78
*p*-Value		0.427	0.563	0.877
Phospholipid (mmol/L): normal range 150~250 mg/dL				
Baseline	225.31 ± 8.88	242.40 ± 7.67	237.90 ± 6.50	229.74 ± 7.13
Follow-up	229.38 ± 8.56	241.85 ± 6.53	246.81 ± 6.78	226.17 ± 7.56
CFB	4.06 ± 5.87	−0.55 ± 6.92	8.9 ± 5.05	−2.11 ± 7.32
*p*-Value		0.812	0.333	0.560

*p*-Value: ANCOVA model with independent variable as baseline and treatment; * *p*  <  0.05 derived from paired *t*-tests performed for values obtained before and after the trial; CFB: change from baseline; TG: triglyceride; HDL-C: high density lipoprotein cholesterol; LDL-C: low density lipoprotein cholesterol.

**Table 8:** Effect of PGE supplementation for 12 weeks on plasma albumin, total bilirubin, alanine aminotransferase (ALP), glutamic oxalacetic transaminase (GOT), glutamic pyruvate transaminase (GPT), blood urea nitrogen (BUN), and creatinine levels in subjects with overweight or obesity (*n* = 100).

	Placebo (M: 15, F: 10)	PGE571 (M: 10, F: 16)	PGE1142 (M: 10, F: 15)	PGE2855 (M: 8, F: 16)
	Mean ± SD	Mean ± SD	Mean ± SD	Mean ± SD
ALB (g/dL): normal range 3.5~5.2 g/dL				
Baseline	4.45 ± 0.04	4.37 ± 0.06	4.37 ± 0.05	4.39 ± 0.04
Follow-up	4.51 ± 0.05 **	4.38 ± 0.05	4.46 ± 0.04 ***	4.42 ± 0.04
CFB	0.06 ± 0.03	0.02 ± 0.04	0.08 ± 0.02	0.03 ± 0.03
*p*-Value		0.080	0.991	0.205
Total bilirubin (mg/dL): normal range <1.3 mg/dL				
Baseline	0.82 ± 0.05	0.73 ± 0.06	0.74 ± 0.06	0.78 ± 0.07
Follow-up	0.84 ± 0.04	0.73 ± 0.06	0.67 ± 0.04	0.76 ± 0.07
CFB	0.01 ± 0.03	0 ± 0.06	−0.07 ± 0.04	−0.02 ± 0.05
*p*-Value		0.438	0.052	0.411
ALP (U/L): normal range 40~160 U/L				
Baseline	56.8 ± 3.20	56 ± 2.23	55.84 ± 2.74	61.08 ± 2.91
Follow-up	57.84 ± 2.99	55.04 ± 2.12	56.08 ± 2.94	59.5 ± 2.99
CFB	1.00 ± 1.37	−0.96 ± 1.80	0.23 ± 1.76	−1.52 ± 2.87
*p*-Value		0.412	0.696	0.608
GOT (AST) (U/L): normal range <40 U/L				
Baseline	32.88 ± 5.10	29.15 ± 3.46	26.68 ± 1.79	26.00 ± 1.87
Follow-up	30.24 ± 4.49	26.92 ± 2.88	23.16 ± 1.13 *	26.75 ± 2.40
CFB	−2.54 ± 3.35	−2.23 ± 4.30	−3.38 ± 1.44	0.72 ± 1.78
*p*-Value		0.668	0.258	0.959
GPT (ALT) (U/L): normal range <40 U/L				
Baseline	31.44 ± 8.21	26.54 ± 3.94	27.08 ± 3.38	24.21 ± 3.06
Follow-up	36.08 ± 9.47	26.46 ± 4.47	21.68 ± 2.25 *	23.17 ± 3.65
CFB	4.46 ± 2.07	−0.08 ± 2.80	−5.19 ± 2.08	−1 ± 2.84
*p*-Value		0.188	0.006	0.121
BUN (mg/dL): normal range 8~22 mg/dL				
Baseline	13.12 ± 0.58	13.31 ± 0.66	12.96 ± 0.49	15.04 ± 0.82
Follow-up	14.24 ± 0.79	12.85 ± 0.66	13.64 ± 0.55	14.08 ± 0.83
CFB	1.08 ± 0.55	−0.46 ± 0.61	0.65 ± 0.50	−0.92 ± 0.78
*p*-Value		0.070	0.550	0.118
Creatinine (mg/dL): normal range 0.5~1.30 mg/dL				
Baseline	0.74 ± 0.03	0.70 ± 0.04	0.70 ± 0.03	0.68 ± 0.03
Follow-up	0.79 ± 0.03 ***	0.74 ± 0.04 ***	0.77 ± 0.04 ***	0.71 ± 0.03
CFB	0.05 ± 0.01	0.04 ± 0.01	0.06 ± 0.02	0.04 ± 0.02
*p*-Value		0.540	0.630	0.365

*p*-Value: ANCOVA model with independent variable as baseline and treatment; * *p*  <  0.05, ** *p*  <  0.01, *** *p*  <  0.001 derived from paired *t*-tests performed for values obtained before and after the trial; ALB: albumin, ALP: alanine aminotransferase, GOT: glutamic oxalacetic transaminase, GPT: glutamic pyruvate transaminase, BUN: blood urea nitrogen, CFB: changes from baseline.
